# Supplementary material for: High-speed and on-chip graphene blackbody emitters for optical communications by remote heat transfer
Source: Nat Commun. 2018 Mar 29;9:1279. doi: 10.1038/s41467-018-03695-x (PMC5876377; doi:10.1038/s41467-018-03695-x)
Supplement: Supplementary file 1 — Supplementary Information (PDF 1148 kb) [file 41467_2018_3695_MOESM1_ESM.pdf]

Supplementary Information

High-Speed and on-Chip Graphene Blackbody Emitters for Optical Communications by Remote Heat Transfer

Yusuke Miyoshi,[†,‡] Yusuke Fukazawa,[†,‡] Yuya Amasaka,[†] Robin Reckmann,[†,§] Tomoya Yokoi,[†] Kazuki Ishida,[†] Kenji Kawahara,[∥] Hiroki Ago[∥] and Hideyuki Maki[†,⊥,]*

[†] *Department of Applied Physics and Physico-Informatics, Keio University, Yokohama 223-8522, Japan.*

[§] *Faculty of Electrical Engineering and Information Technology, RWTH Aachen University, Aachen, 52074, Germany*

[∥] *Global Innovation Center (GIC), Kyushu University, Fukuoka, 816-8580, Japan*

[⊥] *JST, PRESTO, 4-1-8 Honcho, Kawaguchi, Saitama, 332-0012, Japan*

Supplementary Note 1. Emission measurement setups

Emission measurement setups for the high-speed time-resolved measurement and the real-time emission measurements are shown in Supplementary Figure 1a and 1b, respectively.

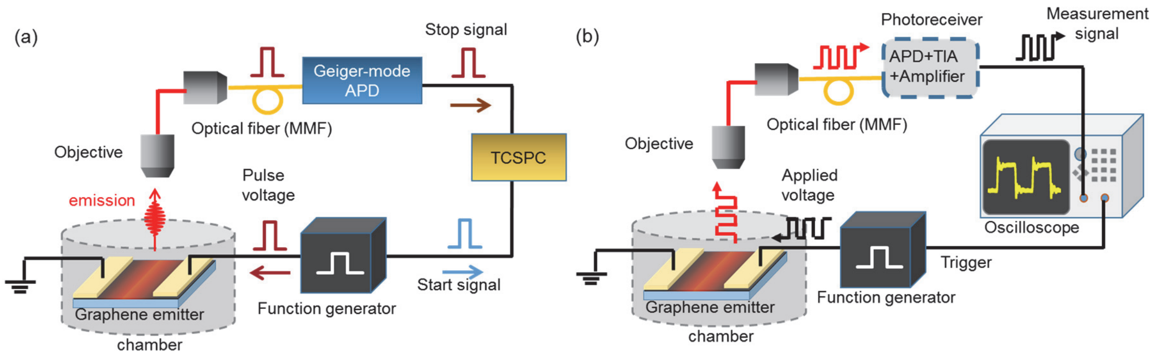

Supplementary Figure 1: Schematic of the time-resolved optical measurement setup. a, The intended voltage pulse is applied as a continuous pulse train. The emitted radiation is guided into a single photon detector (Geiger-mode APD) via an objective and a MMF. A TCSPC module measures the time difference between the applied voltage pulse and the detected photon pulse, and this time differences are plotted in a histogram. **b**, Schematic of the real-time emission measurement setup. A rectangular input is applied to a device, and the emitted light is guided into a conventional photo-receiver, including a normal-mode APD, a transimpedance amplifier (TIA) and amplifier, via an objective and a MMF. The waveform of the measured signal from the photo-receiver is observed by a digital oscilloscope.

Supplementary Note 2. Simulation methods: Transient temperature calculation based on two-dimensional thermal transport in graphene and a substrate

In Figure 3, the temperature response of single-layer graphene is calculated for comparison with the experimental results in Figure 2. The length L and the width W of graphene and the thickness of SiO_2 are 5 μm, 5 μm and 230 nm, respectively. The time-dependent temperature distribution along a graphene emitter can be calculated by solving a one-dimensional heat conduction equation described by

$$C_{\mathrm{GR}}\frac{dT}{dt}=A\frac{d}{dx}[\kappa_{\mathrm{GR}}\frac{d}{dx}T]+p'-q_{\mathrm{Em}}-q_{\mathrm{SPP}}-gW(T-T_{\mathrm{SUB}}),\quad (1)$$

where T and T_{SUB} are the graphene and the substrate temperatures depending on the position of graphene x, respectively. Additionally, C_{GR}, A and κ_{GR} are the heat capacity per unit length (= ρC_{V}, where ρ (≈3.8 × 10^{-12} kg m^{-1} for W = 5 μm) and C_{V} are the linear density and temperature-dependent specific heat of the graphene,[1] respectively), cross-sectional area (1.7 × 10^{-15} m^2 for W = 5 μm) and temperature-dependent thermal conductivity of the graphene,[1] respectively, and p', q_{Em} and q_{SPoPh} are the Joule heating, radiative heat loss rate and surface polar phonon (SPoPh) dissipation rate per unit length, respectively, and g is the thermal conductance to the substrate per unit area. The temperatures of both ends of the graphene and the bottom of SiO_2 are kept at ambient temperature. The temperature-dependent electrical resistivity of graphene is reported in Ref. 2, where the resistivity ρ_{GR} depending on T and carrier density n is given by the sum of the resistivity terms due to the residual resistivity at low temperature ρ_0, the acoustic phonon scattering of graphene $\rho_{\mathrm{A}}(T)$ and the remote SPoPh scattering ρ_{SPoPh} (T, n). The contact electrical resistance between graphene and electrodes is assumed to be 100 Ω[3,4].

To investigate the transient temperature of graphene under a signal input, the thermal transport not only in the graphene but also in the substrate should be evaluated because the substrate temperature strongly affects the transient temperature distribution of graphene. In this study, we solved the simultaneous heat conduction equations for graphene and a substrate, which are combined via the thermal conductance g. The transient temperature distributions for one-dimensional graphene and the two-dimensional substrate are calculated by the finite-difference time-domain (FDTD) method.

In the steady-state blackbody emission in graphene devices, hot spots are formed at the position of the Dirac point due to carrier-density distribution [5-7]. To investigate the effect of the hot spot in the high-speed emission properties, we calculated the transient temperature distribution and the emission intensity, assuming the presence or absence of carrier density n distribution[7].

We elucidate the effect of the remote energy dissipation through the SPoPh scattering in the transient temperature and emission intensity. As reported previously, it has been revealed that the remote SPoPh scattering is the dominant mechanism in the carrier transport on the polar substrate of SiO_2 at high temperature or high electric field[2,8-14]. Since the surface phonon polaritons (SPhPs) excited through SPoPh scattering can propagate in the substrate surface over long distance[15-17], we calculated the device temperature, assuming that the energy loss by SPoPh scattering q_{SPoPh}, which is obtained by the current I and ρ_{SPoPh}, can be directly dissipate outside the graphene device system without the

temperature rise of the substrate beneath the graphene.

The emission intensities of the graphene devices were calculated by the integration of the blackbody-emission intensities along the graphene length, where the emission intensity at each position of graphene was calculated by the integration of Planck's law in the detection wavelength range of the InGaAs APD (0.9–1.6 μm).

Supplementary Note 3. Electrical properties of graphene devices

Supplementary Figure 2 shows the DC bias voltage dependence of the current (*I*-*V* curve) for the emitters with single-, three-, eight-, 28- and 85-layer graphene. *I*-*V* curves of these devices exhibits Ohmic behavior in the low bias voltage range, and these curves slightly saturate in the high bias voltage range due to electron scattering with optical phonons caused by Joule heating[8,10,18-20] . We note that the imperfect saturation of the current might be due to the side-walk current around the hotspot of Joule heating at the center of a graphene.

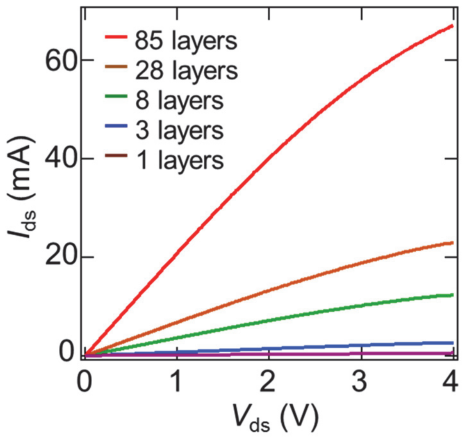

Supplementary Figure 2: I-V characteristics. DC bias voltage dependence of the current for the emitters with single-, three-, eight-, 28- and 85-layer graphene. At relatively high voltages, the current shows a trend of saturation due to phonon scattering.

Supplementary Note 4. Emission properties of the light emitter with three-layer graphene

The light emitters with three-layer graphene also exhibit high-speed light emission as well as the emitters with single-layer graphene, as shown in Supplementary Figure 3.

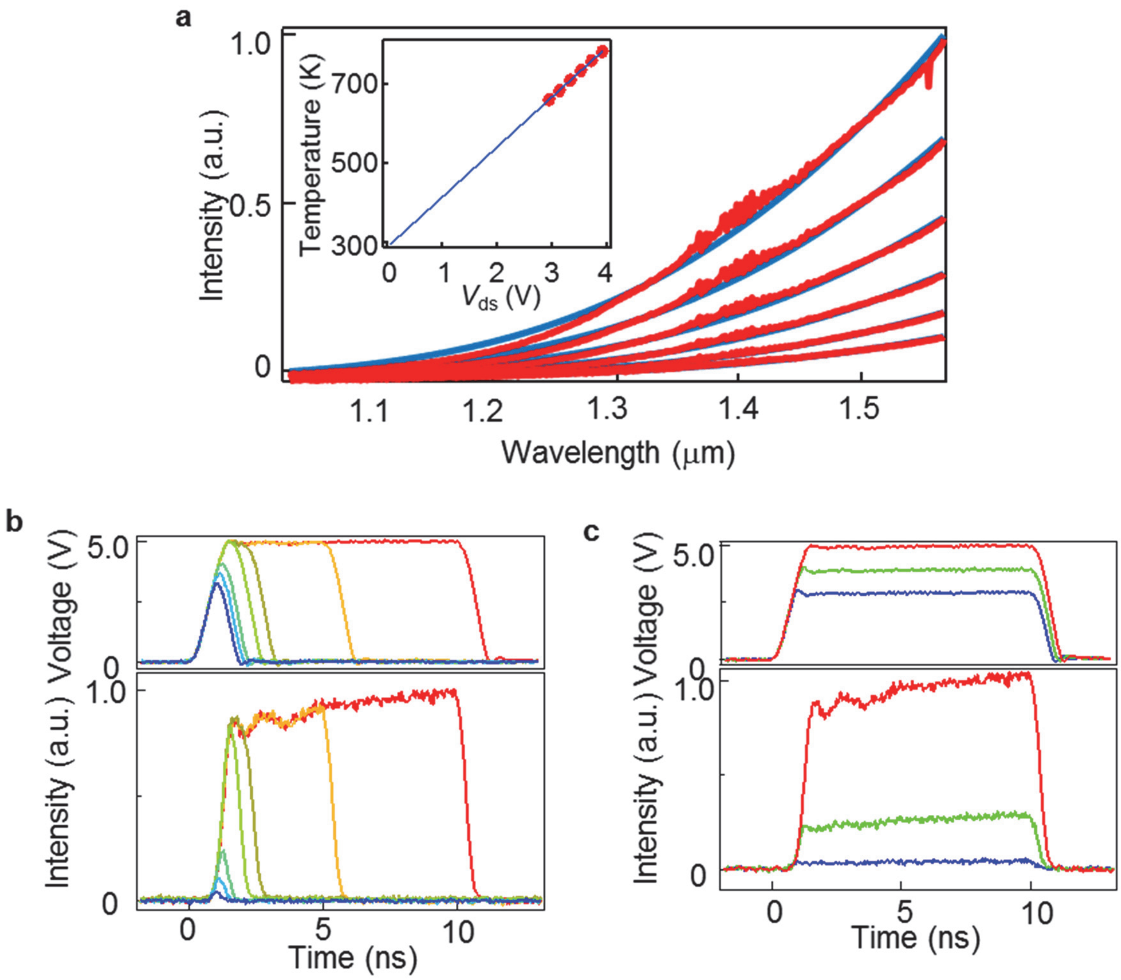

Supplementary Figure 3: Emission properties for the three-layer graphene emitter. a, Emission spectrum of a graphene emitter for V_{ds} = 3 - 4 V with steps of 0.2V for the three-layer graphene emitter. The blue curves are fitting results based on Planck's law. Inset, the temperatures, which is obtained by their fittings, depends linearly on the applied voltages. Time-resolved emission under a rectangular bias voltage with **b** different pulse widths (0.8, 0.9, 1, 1.5, 2, 5 and 10 ns in width and 0 V - 5 V in height) and **c** different pulse amplitudes (10 ns in width and 3, 4 and 5 V in height) from the three-layer graphene device.

Supplementary Note 5. Emisson response of the 85-layer-graphene emitter used in the eye-pattern measurement

In Figure 4b, we demonstrate the optical communicaiton based on the eye-pattern analysis. In this measument, we used very thick multi-layer graphene (85 layers) for higher emission intensity. Taking into account the optical absorption of each graphene layers (2.3 %)[21], the emission intensity increases with increasing the number of grahene layers. Using the emitter with 85-layer graphene, clearly opened eye is observed at 1 Mbps as shown in Figure 4b. We note that the observed eye pattern shows relatively slow asymmetric response. This is explained by the emission response measured by the time-resolved emission shown in Supplementary Figure 4, which can be understood by the slow emission response for the emitter with multi-layer graphene discussed in Figure 2 and 3.

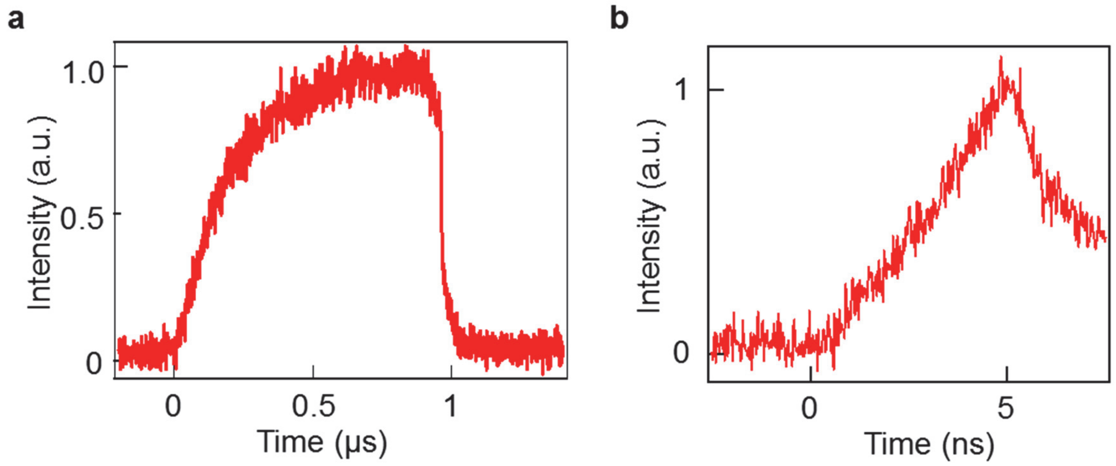

Supplementary Figure 4: Time-resolved measurement for the emitter with 85-layer graphene. Time-resolved emission under rectangular input of **a** 1 μs and **b** 5 ns in width and 0 - 2.4 V in height for the emitter with 85-layer graphene, which is used for an eye-pattern measurement in Figure 4b. The result of **a** can explain the behavior of the observed eye pattern. The result of **b** shows no significant difference from the result of "28 layers" in Fig. 2d, which indicates the time-resolved emissions for "multi-layer" graphene device.

Supplementary References

1. Pop, E., Varshney, V. & Roy, A. K. Thermal properties of graphene Fundamentals and applications. *MRS Bulletin* **37**, 1273-1281 (2012).
2. Chen. J.-H., Jang, C., Xiao, S., Ishigami, M. & Fuhrer, M. S. Intrinsic and extrinsic performance limits of graphene devices on SiO2. *Nat. Nanotechnol.* **3**, 206-209 (2008).
3. Xia, F., Perebeinos, V., Lin, Y., Wu, Y. & Avouris, P. The origins and limits of metal-graphene junction resistance. *Nat. Nanotechnol.* **6**, 179–184 (2011).

4. Robinson, J. A. *et al.* Contacting graphene. *Appl. Phys. Lett.* **98**, 53103 (2011).
5. Freitag, M., Chiu, H.-Y., Steiner, M., Perebeinos, V. & Avouris, P. Thermal infrared emission from biased graphene. *Nat. Nanotechnol.* **5**, 497-501 (2010).
6. Bae, M.-H., Ong, Z.-Y., Estrada, D. & Pop, E. Imaging, simulation, and electrostatic control of power dissipation in graphene devices. *Nano Lett.* **10**, 4787-4793 (2010).
7. Bae, M-H., Islam, S., Dorgan, V. E. & Pop, E. Scaling of high-field transport and localized heating in graphene transistors. *ACS Nano* **5**, 7936-7944 (2011).
8. Meric, I., Han, M. Y., Young, A. F., Ozyilmaz, B., Kim, P. & Shepard, K. L. Current saturation in zero-bandgap, top-gated graphene field-effect transistors. *Nat. Nanotechnol.* **3**, 654-659 (2008).
9. Fratini, S. & Guinea, F. Substrate-limited electron dynamics in graphene. *Phys. Rev. B* **77**, 195415 (2008).
10. Rotkin, S. V., Perebeinos, V., Petrov, A. G. & Avouris, P. An essential mechanism of heat dissipation in carbon nanotube electronics. *Nano Lett.* **9**, 1850-1855 (2009).
11. DaSilva, A. M., Zou, K., Jain, J. K. & Zhu, J. Mechanism for current saturation and energy dissipation in graphene transistors. *Phys. Rev. Lett.* **104**, 236601 (2010).
12. Li, X., Kong, B. D., Zavada, J. M. & Kim, K. W. Strong substrate effects of joule heating in graphene electronics. *Appl. Phys. Lett.* **99**, 233114 (2011).
13. Koh, Y. K. *et al.* Role of remote interfacial phonon (RIP) scattering in heat transport across graphene/SiO_2 interfaces. *Nano Lett.* **16**, 6014-6020 (2016).
14. Serov, A. Y., Ong, Z.-Y., Fischetti, M. V. & Pop, E. Theoretical analysis of high-field transport in graphene on a substrate. *J. Appl. Phys.* **116**, 034507 (2014).
15. Chen, D.-Z. A. & Chen, G. Measurement of silicon dioxide surface phonon-polariton propagation length by attenuated total reflection. *Appl. Phys. Lett.* **91**, 121906 (2007).
16. Ordonez-Miranda, J. *et al.* Anomalous thermal conductivity by surface phonon-polaritons of polar nano thin films due to their asymmetric surrounding media. *J. Appl. Phys.* **113**, 084311 (2013).
17. Chen, D.-Z. A., Narayanaswamy, A. & Chen, G. Surface phonon-polariton mediated thermal conductivity enhancement of amorphous thin films. *Phys. Rev. B* **72**, 155435 (2005).
18. Berciaud, S., Han, M. Y., Mak, K. F., Brus, L. E., Kim, P. & Heinz, T. F. Electron and Optical Phonon Temperatures in Electrically Biased Graphene. *Phys. Rev. Lett.* **104**, 227401 (2010).
19. Freitag, M. *et al.* Energy dissipation in graphene field-effect transistors. *Nano Lett.* **9**, 1883-1888 (2009).
20. Liao, A. D. *et al.* Thermally limited current carrying ability of graphene nanoribbons. *Phys. Rev. Lett.* **106**, 256801 (2011).
21. Nair, R. R. *et al.* Fine structure constant defines visual transparency of graphene. *Science* **320**, 1308 (2008).
